# Lecture, Introductory, to the Study of the Principles and Practice of Medicine

**Published:** 1849-09

**Authors:** Austin Flint

**Affiliations:** Prof. of Principles and Practice of Medicine in the Medical Department of the University of Buffalo


### BUFFALO MEDICAL JOURNAL

AND

### MONTHLY REVIEW.

VOL. 5. SEPTEMBER, 1849. NO. 4.

### ORIGINAL COMMUNICATIONS.

ART. I.—*Lecture, Introductory, to the Study of the Principles and Practice of Medicine.* By Austin Flint, M. D., Prof. of Principles and Practice of Medicine in the Medical Department of the University of Buffalo.

[The following discourse was read at the commencement of the course of instruction in the Principles and Practice of Medicine, in the Medical Department of the University of Buffalo, session of 1848–9. As the topics and considerations presented (it may, perhaps, be presumed,) possess some interest for the medical practitioner, as well as the student, and as they are treated in a manner suited rather for perusal, than oral delivery, we venture to give the discourse insertion, although not written with the least view to publication. It is proper to add, that the idea of its publication, probably, would not have occurred to us, were it not for a dearth of other original communications, at this time, when most of those upon whom we chiefly depend for contributions to our pages, are absorbed in the practical duties incident to the prevailing epidemic. The latter consideration is offered as an apology for converting a *lecture* into an *article,* should an apology seem to any of our readers to be required.]

Gentlemen:—Before engaging in any undertaking which calls for a considerable expenditure of time and labor, it is the part of wisdom and prudence to make oneself acquainted with the objects and duties which the undertaking involves. Such a preliminary survey, it may be reasonably expected, will tend, in no small degree, to facilitate subsequent exertions, and promote success, by enabling us to direct our efforts in a more systematic and efficient manner than if we were to commence precipitantly, without a clear understanding of the specific ends to be fulfilled, and the best course for their fulfilment. In accordance with this policy, before we enter upon the course of instruction in which we are about to engage, I will invite your attention to some considerations relating to the character and scope of the subjects which our teachings will embrace, and to some of the leading aims which it were well should be kept in view in the prosecution of our labors.

The departments of medical science assigned to the chair which I have the honor to occupy in this Institution, are thus designated: — "The principles and practice of medicine, and clinical medicine." The phrase, clinical medicine, is understood to apply, strictly speaking, to instruction given at the bed side of patients—in other words, agreeably to the etymology of the term '*clinical*,' *bed-side* instruction—but, by a constructive signification, the phrase comprehends any instruction which relates to cases of disease under actual observation, i. e., in which the patient is seen and examined by, or before the class. The phrase, "demonstrative medicine" is, of late, sometimes employed to denote this important branch of medical education. It will be my duty, as teacher of clinical medicine, to endeavor to make available for your instruction whatever cases may present themselves at the College, and at the Hospital to which you will have access. The arrangements and plan which will be followed in this division of my course, I will reserve for remark on some other occasion.

The "*principles and practice of medicine*" — let us inquire what subjects fall legitimately in the division of medical instruction thus designated. The subjects which this branch of medical study, embraces, relate constantly, and directly, to *Disease*. This fact serves to distinguish this division from some of the departments into which medical instruction is divided. Anatomy, Physiology, Chemistry, concern subjects which, altho' essential as pre-requisites for the study of disease, yet, have not immediate and exclusive reference to it. We shall find it necessary to adduce facts appertaining to each of the branches just named, in the elucidation of the *principles and practice of medicine*, but it is obvious that these pursuits would admit of being prosecuted if disease did not exist. They *must* be pursued in order to be prepared to study disease, and they recommend themselves to us, chiefly, with reference to the study of disease, but they have no dependence upon the latter study, and *may* be pursued wholly irrespective of it. The remainder of the several branches into which medical instruction is usually divided, are not, like those just named, distinguished by the comparative remoteness of their relations to disease. On the contrary, some of them concern disease nearly, if not quite as intimately as do the *principles and practice of medicine*, and some of them, as we shall hereafter find, are, to a considerable extent, involved in the latter pursuit. The principles and practice of surgery, which constitute a distinct division of study, involve disease nearly to the same extent as medicine proper, its characteristics, as a distinct department, consisting in its restriction to diseases situated externally, and also to the fact that, with, or without medical treatment, the knife, and various other appliances of a mechanical nature, are called into requisition. Midwifery, another separate branch, involves, first, physiological, not morbid functions, of which medical supervision is generally necessary, viz: the functions of conception, gestation, and labor. The various accidents, aberrations of function, and surgical measures incident to these functions, are naturally included in the same branch of instruction; and, also, by conventional agreement, the diseases peculiar to females and children. Both surgery and midwifery are properly branches of the principles and practice of medicine, in the comprehensive sense of this expression; and, in this country, the three departments are usually united in the medical practitioner. They are separately taught, to secure the advantages of division of labor, as respects both the teacher and pupil, and this division of labor is useful when carried to some extent into practice.

Forensic Medicine, a highly interesting and important division, also involves, in a considerable degree, knowledge of disease, but disease considered in a particular aspect, to wit: in its relation to jurisprudence or civil law. Materia Medica and Pharmacy, also, directly relate to disease, but to disease considered relatively to those agents, and their preparations, which exert a remedial action upon the economy.

I have thus noticed all the divisions of medical instruction agreeably to the arrangements of this Institution, and of medical institutions generally, exclusive of the *principles and practice of medicine*, with a single exception. The excepted branch is *General Pathology*. This branch is more closely allied to the principles and practice of medicine than any of the others. Indeed, *General Pathology* and *principles of medicine* are expressions almost convertible, the subjects embraced by both being nearly identical.

But, as separate branches of instruction, there exists a distinction between them, which I will endeavor to explain, in order that the boundaries of the province assigned to me may be more clearly defined. In order to appreciate the distinction, it is to be remarked, in the first place, that the term disease, as used in medical writing and conversation, has not always the same latitude of signification. We are accustomed to apply the term disease, in a very general sense, to denote the fact of a morbid condition, without any reference to its character or seat. We say a person is suffering from disease; then the question arises, what disease does he labor under? This question may still be considered to apply to disease, considered in a general or generic sense. For example, we may say in reply, that the disease is inflammation, without specifying the particular character and location of the inflammation. We call inflammation a disease, and so hemorrhage, irritation, &c., are other diseases. On the other hand, the term has a more specific meaning when we state the precise seat and kind of disease. For example, when a person is said to have inflammation of the brain, encephalitis, or hemorrhage into the brain, apoplexy, etc.

Now, *general pathology*, as a branch of medical instruction, treats of disease limited, in its signification, to its *general* or *generic* sense, not considering it in its special or particular applications. The latter belong to the *principles and practice of medicine.* Hence, the significancy of the expression *general* pathology, while the phrase *special pathology* may be, and is applied to express the study of the special forms of disease, or, in other words, individual diseases. General principles appertaining to disease in its most general sense; and to its generic divisions; the general doctrines of the causes of disease (or etiology), of symptoms (or semeiology), of discrimination between different diseases (diagnosis), &c., furnish subjects which belong to general pathology. The principles and practice of medicine, or special pathology, on the other hand, deals with these same principles and doctrines considered in their applications to disease, as it is presented in particular locations, in its special forms, and varieties, and in individual cases.

We might characterise general pathology as the philosophy of medicine, while the department upon which we are about to enter, teaches the *philosophy* in its applications to the *art* of healing. General pathology does not go beyond principles, except for illustrations of principles, but the principles and practice of medicine, as the expression itself signifies, considers principles with constant reference to practice. The same division of subjects will be found to obtain in every department of knowledge which embraces both a science and an art, but, to select a comparison which will be most familiar to you, general pathology bears a relation to the principles and practice of medicine, analogous to that subsisting between general and special anatomy, the subjects of the former being, as you know, the elementary structures, or tissues, into which the organism is capable of being resolved, while the latter pursuit, i. e., special anatomy, describes the apparatuses and organs formed by these elementary structures, compounded in various proportions.

I have dwelt somewhat upon the distinction which I have just endeavored to explain, because it is desirable, in itself, for the student to have a clear apprehension of it, and because he will be better prepared to understand the scope of inquiry legitimately belonging to the instructions emanating from this chair. While there are many things which belong in common to both departments, it will be perceived, (if I have succeeded in making the distinction between them intelligible,) that my learned colleague to whom the department of general pathology has been assigned, will be entitled to a range of inquiry and discussion with regard to various points of scientific interest and importance, which are beyond the limits of the course of instruction to which these remarks are prefatory. The proper field which belongs to us, respectively, will be rendered more clearly apparent when we come to pass, in prospective review, the several classes of topics, which, in the prosecution of our labors, will engage our attention.

I have said that the division of medical science upon which we are about to enter may be characterised by its constant, proximate reference to *disease*. We have just considered some differences as respects the latitude of signification, appertaining to the common acceptation of disease. Let us, for a moment, direct our attention to some farther considerations connected with the term. All the information which it will be my endeavor to assist you in acquiring in the course of my instructions, will relate to the province of knowledge denoted by this term *disease*. Now, in entering upon any province of knowledge, one of the first objects of inquiry is, the specific nature of the knowledge which we seek to inquire. What, then, we may ask, is disease? Has it ever occurred to you, gentlemen, to attempt to define the term? If you will reflect for a moment upon this inquiry, you will perceive, that its answer involves some difficulties. It is not easy to express in positive, exact terms, a definition of disease. The term does not designate any single entity, but it comprehends various conditions, and numerous phenomena. In fact, we can only define it by negation — that is to say,

*disease is the absence of health.* To understand in what disease consists, then, we must previously take into consideration what is essential to health. Now, observation and experience teach us that for a person to be in a state of health, the organs of the body necessary to the safety and welfare of the organism, and to the comfort of the individual, must be sound, and their functions well performed. It is not requisite that the body shall preserve its entire integrity. A man may lose a leg, or an arm, and enjoy perfect health, the extent of his physical capacities for enjoyment being alone curtailed. So, also, every trifling aberration of function does not necessarily constitute disease. We do not say when we suffer a slight headache, or experience uncomfortable sensations from imprudence in diet, or fatigue from over-exertion of body and mind, that we are laboring under disease. It is only when the natural play of organs and functions, and personal ease, are compromised beyond such trivial and transient derangements, that the term disease becomes properly applicable.

I may remark here, in passing, that the term *disorder* is frequently used to denote certain disturbances in the economy, falling short of that amount necessary to constitute *disease.* The two terms have each a distinctive sense, which, although not capable of being very exactly represented by words, is, nevertheless, sufficiently understood. It is difficult to say with precision what amount of disturbance ceases to be properly a *disorder*, and becomes a *disease*, but, for all practical purposes, the distinction is sufficiently appreciable. Disease, then, is a term corelative to health, involving, by necessity, the latter, although the latter term does not reciprocally involve the former; and it does not abstractedly admit of being rigorously defined.

Let me, now, gentlemen, direct your attention for a few moments to the *generic or elementary forms of disease.* You will please call to mind the two-fold signification of the term disease, of which I have already spoken. In one signification, as you will recollect, it applies to those conditions deviating from health, (i. e., morbid conditions,) which are distinguished from each other by intrinsic differences. *Inflammation, irritation, congestion,* &c., wherever situated in the body, and without reference to other variations, are instances of disease considered in this sense. These are, in other words, the generic or elementary forms of disease, into which all the phenomena of disease are resolvable. In the second signification, the term is applied to the special manifestations of disease, as when inflammation, irritation, congestion, and other elementary forms of disease are situated in particular parts, and otherwise divided by specific distinctions. The latter are the special manifestations of disease, or, in other words, special diseases. In the former sense, disease is considered *pathologically*, in the latter *nosologically*. but the force of this distinction will better appear hereafter. The *elementary forms of disease*, as we have seen, are the proper subjects of *general pathology*, while *special diseases* belong to us. We cannot, however, enter upon the latter without some notice of the former, and in the remarks which I shall devote to this subject, my object will be, simply to premise a few considerations whieh are appropriate as introductory to the *principles and practice of medicine*, without encroaching on the province of instruction belonging to my learned colleague, the Professor of general pathology.

The special manifestations of disease, or special diseases, as we shall hereafter see, are numerous. On the other hand, the elementary forms of disease are few in number. We obtain our list of elementary forms of disease, as we do other classified facts in science, by the logical processes of generalization, We analyse the phenomena of disease, compare them with each other, and arrange them, from their resemblances, into as few classes as possible; and the degree of perfection to which this generalization can be carried, depends greatly on the means of analysis and comparison which, in the present state of science, are at our disposal. In the study of the phenomena of disease, as in the study of other natural phenomena, to generalize into the simplest elements into which these phenomena are capable of being resolved, is a prime object of scientific investigation. These elements stand in the relation of physical causes to the various phenomena which present themselves to our observation, in the same light that affinity is the cause of chemical phenomena, gravitation, cohesion, etc., of the phenomena of physics. Of the philosophical methods by which the processes of generalization are effected, and their results obtained, you are presumed to be acquainted.

No generalization which can now be made is perfect, or will be permanent. The process of science, by developing new analogies, may lead to the reduction of some form of disease which are now regarded as simple, into different, and still simpler elements; or, on the other hand, may unite together into a single element, two or more elements now regarded as separate. With reference to the number of the simplest forms of disease, different teachers will differ, which, in itself, suffices to show that generalization with our present knowledge, is necessarily imperfect. I shall proceed to name the several classes, which, in my view, will embrace all the elementary forms of disease, as we are at present bound to consider them.

1st. *Inflammation.*—This term, by universal consent, is considered to express an ultimate element of diseased action. The history of inflammation, its characteristic phenomena, the varieties of its manifestations, the conditions of its occurrence, its causes, its modifications, its results, and its essential nature are topics of great interest and importance. To treat of these topics formally, does not fall within my province, but they will be found to enter largely into the consideration of special diseases, and in that connection, it will be necessary for us to treat, incidentally, of the more important practical principles belonging to the subject.

2d. *Fever.*—By most, but not by all, *fever* is deemed to be an elementary form of disease. We shall consider the propriety of so viewing it, hereafter.

3d. *Irritation.*—A morbid condition of the solid textures entitled irritation, is generally regarded as elementary, i. e., resolvable into no other condition. It is clearly allied to inflammation, yet differs from it in not presenting those visible, appreciable phenomena and results, which are characteristic of the inflammatory state. It consists, apparently, in a morbid condition of the dynamic, or vital endowments of parts, and is generally supposed to involve, particularly, the nervous constituents of the affected tissues.

4th. *Congestion.*—This may be regarded, properly, as an elementary morbid condition. Very generally, however, it is traceable to other anterior, or coincident conditions of disease, of which it is only an effect, and, under such circumstances, not elementary. Nevertheless, as it does occur when it cannot be referred to any affection which stands to it in the relation of a cause, it is to be included in the list of elementary affections.

5th. *Effusion and Hemorrhage.*—These are also almost always merely results, or effects of pre-existing or co-existing disease. But as they cannot always be said to be in that way contingent, they are to be considered, for the present, at least, as occasionally elementary.

6th. *Morbid Assimilation.*—This is a very comprehensive expression, and will embrace a great number of phenomena, of the essential nature of which, we, at present, know but little. Under this head are to be included all the morbid changes which take place in the blood, from aberration of any of the processes which contribute to form that fluid, or from the introduction of poisonous principles into the circulation. Limited as is our knowledge of the disorders arising from this source, enough is known to afford wide scope for pathological discussions.

General principles deducible from the knowledge already possessed of the changes of the blood arising from morbid assimilation, will enter, in no inconsiderable degree, into the history of special diseases.

7th. *Morbid Innervation.*—This class embraces disorders affecting the nervous system. The title, as you perceive, is merely a generic expression, and does not denote any well defined deviation from a healthy state. But, as we shall have occasion to see when we come to treat of diseases of the nervous system, we are, at present, unable to explain the essential conditions upon which they depend. In our generalization, therefore, we must content ourselves with distinguishing them, collectively, by a title, and *morbid innervation*, as it involves no hypothesis, will answer as well as any mode of expression.

8th. *Morbid Elaboration.*—In this class I would include affections of nutrition, secretion, transformations of texture, and new or heterologous deposits.

Not to be misunderstood, let me repeat, emphatically, that I do not present this enumeration of elementary affections with any degree of confidence that it actually expresses radical divisions into which diseases are capable ultimately, or intrinsically, of being resolved; but only those which are incident to the present condition of Medical Science. It is, in fact, open to objections even with our present knowledge. Some of the classes, last mentioned, morbid innervation, assimilation, and elaboration, obviously enough embrace several affections, which analysis, even now, perhaps, could resolve into different elementary diseases; but as it is desirable not to multiply divisions further than our actual knowledge authorizes, I have endeavored to avoid extending the classification, so as to incur the liability to hypothetical assumptions.

It has always been a fundamental error in medical speculations, (and it is one which, at this day, the student and physician cannot too much guard against,) to endeavor to generalize primary morbid conditions into one or two ultimate principles. Thus, to go back only a half century, the Brunonian doctrine, so called from its ingenious author, endeavoured to reduce all diseases into a *plus* and *minus* quantity of a certain suppositious force called *excitability*. Life, according to that theory, is the result of a certain reaction of excitability to various internal and external influences, called stimuli. Health consists in a proper balance between the stimuli and the excitability. Disease consists in either an excess, or diminution of excitability. The excess gives rise to diseases called *sthenic;* the diminution to those called *asthenic*, (these terms, as will be observed hereafter, are sti in use, in a figurative sense). Since it is much more easy, on this hypothesis, to account for the loss, than the gain of excitability, it followed that nearly all diseases were those of debility, and that the appropriate remedies for nearly all diseases were stimulants. This doctrine, rendered attractive by its plausibility and simplicity, prevailed, for a considerable period, to a great extent. Even now, as we are informed, it is inculcated at some of the Italian schools.

Still more recently the theory of Broussais, which reduced all diseased action to inflammation, and localized all those affections which had been supposed to consist in a general disorder of the economy, furnishes another instance of the same delusive tendency to erroneous generalization, I can personally bear witness to the fascination of this doctrine, which resolved the principles and practice of medicine into a few plain and simple formulas.

To illustrate the absurdity and the evils of such attempts, it is only necessary to contrast the two doctrines just alluded to, with their practical consequences. While Brunonism led to the almost universal application of stimulants as remedies; Broussaisism, on the other hand, denounced stimulants, in all cases, as incendiary, and advocated general and local depletion as the therapeutic principle in nearly all diseases ! The extent to which both dogmas prevailed, goes to show most conclusively, that experience, and observation of results, furnish by no means an infallible, or, at all events, a readily available antidote to errors of speculation.

It is, at the present day, considered the part of wisdom and prudence to abandon a project in which the efforts of distinguished genius have thus far signally failed. The course to be pursued, albeit less attractive to an ambitious mind, is plain, and in its results far more satisfactory. We must generalize, in the science of disease, as in other natural sciences. We are to study facts which have important bearings, whether derived from Physiology, the phenomena presented during disease, or autopsical researches. By careful comparison, we are to resolve morbid affections into as few single elements as possible. The elements remaining after the analysis has been carried as far as the state of Science will allow, are to be regarded as the simplest form of disease, which, as we have said, it is the peculiar province of general Pathology to elucidate.

With regard to the now considered primary or original forms of diseased action, it is altogether probable that we are somewhat in the same situation as was the scientific world, many years ago, with reference to principles of other sciences. The ancients thought that some bodies possessed a specific levity, and it is not long since the ascent of liquids in hydraulic tubes to find their level, was attributed to a peculiar principle called "Nature's abhorrence of a vacuum." So, at some future day, we shall perhaps look back upon the present classification as, in many respects, not only erroneous, but absurd, when contrasted with the few, simple, and intelligible elements into which successful scientific researches, and philosophical investigations have resolved pathological phenomena. This we can readily conceive of, and the reflection may very properly serve to stimulate zeal, and promote the active employment of all legitimate means for the advancement of science ; but we have no right to derive from it any authority to substitute mere speculation in the place of logical inductions from established facts. The latter must satisfy us, although they may fail to realize all our expectations, and satisfy our wishes.

It is by no means to be expected that scientific inquiry will stop short at the several elementary forms of disease which have been submitted. The questions cannot but arise, what is Inflammation ? what is Irritation ? and so of the others. It will not be expected of me to enter upon any discussion of these questions. I adduce them simply to make some further remarks on the discrepancy of doctrine which not only has existed in former times, but which is still exemplified by the different opinions of medical philosophers. I do not design, however, to enlarge upon this subject, but to give some exposition of the views which will actuate me in my course of instruction in special pathology.

Disease, as we have said already, can only be defined by saying it is a deviation from a healthy condition of the organism. Health involves certain normal conditions of the solid and fluid constituents of the body. Now, in which of these constituents is the point of departure for disease? Does disease generally involve, primarily, the solids or fluids? This inquiry brings us to a point which has given rise to much discrepancy of doctrine, and which is still a subject of discussion. Does disease always, or ordinarily depend upon a morbid action of the fluids? The affirmative to this question was the ancient doctrine, and was maintained for many centuries. It constitutes what is called the "humoral pathology." When the physical sciences came to be studied with such success, in modern times, the humoral pathology was abandoned, and disease, in nearly all instances, was supposed to originate in the solids. Hence arose the school of the *solidists,* in distinction from that of the *humoralists.* The pathological researches of Hunter, Bichat, and their successors, which were confined especially to the morbid changes of the tissues, tended to concentrate the attention of medical philosophers upon the solid parts, and exclusive solidism, for many years, was the predominant doctrine. Of late, the application of chemistry to the analysis of the fluids in health and disease, has produced a sensible diversion in the current of medical opinions. The majority of medical men are perhaps still, to a great extent, *solidists*. A large proportion, however, and these are more apt to be among the junior members of the profession, attach great importance to the fluids. Any one who has observed the medical history of the last fifteen years, must perceive that the direction toward humoral views has been constantly increasing, and that the latter appear to be fast predominating.

As I have remarked, it cannot be expected that I should engage in an elaborate discussion of this subject. In treating of special diseases, I shall present such views of their essential character as shall appear to me, in the present condition of science, most rational. But I wish to say here, that I do not feel authorised to teach any creed, or set of doctrines, with regard to the subject, nor would I advise you to limit yourselves to any exclusive views. The best course for the student, and the practitioner, on this, as on all points respecting which there are conflicting doctrines, is to endeavor to consider fairly, and without prejudice, the evidence on all sides, to avoid premature conclusions, and in cases in which it is difficult to reconcile conflicting facts, to await patiently farther developments of science. *Suspension of judgment* is far preferable to opinions hastily and crudely adopted. He is much nearer the truth who has delayed arriving at positive conclusions, than he who has fallen into error; for, in the latter instance, false steps are to be retraced, and we all know that it is much more difficult to unlearn, than to learn. Besides, a person who has once committed himself as a partizan to certain opinions, is liable to be restrained by self-love from exercising that impartial examination of facts pertaining thereto, which is essential to a truly philosophical discrimination of truth.

In view of differences of doctrine relating to the origin and essential character of morbid actions, the position which appears to my mind to comport best with what knowledge is actually possessed, is that of a *rational eclecticism*.

That disease may be induced by a primary change exerted upon the fluids, seems to me demonstrable. For example, mercury received into the stomach in such fractional quantities as to occasion no appreciable effect upon the solids, is absorbed by a natural, not a morbid, process, into the circulation, and produces certain specific diseases, the most common of which is Ptyalism, or salivation. It may be said that the poison, in this case, is simply taken into the circulation, and transported to the salivary glands, where, unchanged in its properties, it exerts its effects upon the solid parts: but it is altogether more reasonable to suppose, that there is some change exerted upon the sanguiferous fluid, and what confirms this is, that various other effects than Ptyalism may be produced—for instance, a change of action in parts already diseased, a change which mercury, if directly applied, would not induce. It is probable that those diseases which are produced by morbific agents, such as the virus of contagion, malaria, &c., are, to a certain extent, analogous in their origin to the artificial diseases which may be induced by mercury and other poisonous substances.

On the other hand, in some diseases, the primary morbid change in the series appears to be limited to the solids. Inflammation, for example, would seem to be thus situated. It is true, that in many, if not in most diseases, the fluids become perverted, but this is perhaps an effect of a prior perversion of the solids, so that the latter may be primary, although various consecutive evils may arise from the perverted condition of the fluids.

In treating of particular diseases, it will be a point of inquiry, in every instance, to ascertain whether the fluids or solids are primarily, or chiefly involved. The inquiry, in my opinion, is to be met, not with any predetermined creed or doctrine, but by an impartial examination of the facts bearing upon each particular instance. Nor are we to refuse to admit facts from whatever source they may be derived. If chemistry furnish them, supplanting agencies which we have heretofore attributed to a vital force, we are not to turn them aside with the scornful charge of heterodoxy. Science has no creeds, no tests of faith, but she desires truth, and truth only. Let it come whence it may, its claims are to be considered paramount and inviolate!

There is much to be said, Gentlemen, on the dangers of exclusiveness, ultraism, and partizanship, in the formation and advocacy of medical opinions. I cannot, however, on this occasion pursue the subject farther. I have introduced it merely that our preparatory reflections may enable us to enter upon the study of diseases with a candid, philosophical spirit. Let us undertake our investigations with minds divested of all prejudices, or bias toward any particular speculative views—let us not call ourselves "*humoralists*," nor "*solidists*," "*vitalists*" nor "*chemicalists*;" but only honest seekers after true knowledge, in so far as it is attainable with our limited apprehensions, and in the present condition of our science.

In preparing for the acquisition of scientific knowledge, and, at all times, in the prosecution of scientific pursuits, it is important to meditate on the means by which truth is to be investigated, so that among the facts which are communicated to us, or developed by our own investigations, we may be able to discriminate between those which have been absolutely or demonstratively established, and those which are merely hypothetical. Here opens a field of reflection affording wide scope for discussion, and much room for profitable counsel. I must content myself, however, with a few remarks.

The principles and rules which are to regulate medical reasoning are, of course, identical, in the main, with those presiding over the investigations of every other class of natural phenomena. Yet, the application of logical methods to the study of disease has given rise to some discrepancy of doctrine.

The evidence for the facts of medical science is of two kinds, consisting 1st., the direct results of experience; 2d., in rational deductions from already established facts. Facts resulting exclusively from experience are often entirely unintelligible. We know them to be facts, simply because, experience declares them to be so. We cannot gainsay her assertions, but we are unable to account for them by their relations with other facts, or by means of any known laws. For example, Quinia will cure fever and ague; observation has abundantly established this fact, but science does not enable us to explain the *methodus operandi* of the remedy, by which the cure is effected. We must be content, for the present, to accept the bare fact, without knowing its *rationale.* This is a familiar example taken from therepeutics, but numerous illustrations may be cited from every department of medicine. These facts are often distinguished as "*Empirical facts.*" On the other hand, we are often able, deductively, to determine important facts; reasoning from acknowledged premises, and arriving at certain conclusions in anticipation of the results of experience. To select, at random, a plain and striking case in which an analogical deduction would be admissible; if we know that a person is suffering from acute gastritis, we may reasonably infer that it will be injudicious to administer an active stimulant, or acrid medicament, even without being aware that the experiment had been ever been tried. We should reasonably infer from our manifold observations with respect to inflammation in localities where it can be seen, and the effects of applications witnessed, that a highly inflamed mucus membrane requires soothing, rather than irritating remedies. Now, this is a deductive, or analogical conclusion; and the fact may be distinguished as a "*rational fact.*" We not only believe it to be a fact, but we can give a good and sufficient reason therefor. The cases are not infrequent, in the practice of medicine, in which we are not only authorised, but compelled to form and act upon rational facts. Yet the position has been deliberately assumed by a distinguished American writer and teacher, that Empirical facts only are worthy of reliance; that the evidence of experience is alone admissible for the induction of principles of pathology and therepeutics; that the experimental is the only legitimate method of investigation in medical science. Time will not permit me to dwell upon this topic sufficiently to adduce considerations to illustrate its untenableness, and, indeed, I may say, its absurdity. It is an instance of the ultraism and exclusiveness to which enthusiastic minds are prone, without the exercise of great circumspection. That experience is the great test of truth, the tribunal from which there is no appeal, will be conceded, but that medical science can advance no further than it is led by experimental knowledge, is to lay down a rule which must be practically broken daily by those who enunciate it. Abundant occasions will present themselves in treating of the various classes of disease to show how valuable have been the practical deductions from new discoveries in physiology and pathology, and how much Medicine is indebted to rational facts. Let me not be understood to disparage the value of experience, or of empirical laws. I concede, to the fullest extent, their importance, but deny that they are to supersede the deductions of ratiocination. Reason and experience, indeed, always should go hand in hand in the pursuit of scientific knowledge. They are not only twin brothers, but, like the Siamese twins, they are bound together by a connecting bond, which, to sever, would be to destroy both.

Much has always been said concerning Medical experience. It is well to ask ourselves in what experience consists. I cannot, on this occasion, follow out fully the train of thought which this question suggests. I can only offer a few discursive remarks with a view to the direction of your own reflections. We often hear complaints of the number of "false facts," as they have been significantly called, which prevail in medical science. Now, whence are these "false facts" most apt to spring:—from theoretical deductions, or from erroneous experience? It is clear to my mind that the latter is by far the more prolific source. Rational errors are more easily detected, and meet with greater difficulty in acquiring currency; but to disprove the false results of experience, a tedious process is often necessary, and it not infrequently happens that a long period elapses before the error is detected. A close examination of the conditions necessary for the determination of accurate empirical facts, and the many liabilities to erroneous results in medical observations, must satisfy every reflecting mind, that it is by no means an easy process, nor an universal faculty. If you look around upon the medical men of your acquaintance, how many of them possess capabilities which authorise you to rely, with implicit confidence, on the fruits of their experience? Ascertain and compare their peculiar opinions; consider how greatly they often differ, yet they are all confidently based upon their individual experience! Experience, then, is not an uniform, infallible method of discovering truth, as practiced by the profession at large. Look, also, into the different opinions which, at different periods, have been accredited and adopted by the profession at large. Experience has been, professedly, at the foundation of them all! In fine, experience, unless directed by reason, prudence, and skill, is a most fallacious guide; and its errors are the more serious, not only because they are often difficult to be disproved, but because it is too often supposed to be infallible.

The requisitions of true experience, the observances and precautions which it imposes, claim, in an emphatic manner, the close study of the medical student and practitioner. One of the most distinguishing characteristics of modern medicine may be said to consist in the more philosophical application of experience to medical investigations: and for this our Science is greatly indebted to the precepts and example of the distinguished French pathologist, M. Louis, and his followers. To Louis, as it seems to me, is justly due the credit of having, more clearly than any previous medical philosopher, discriminated false, from true experience, and resolved it into a truly philosophical system. Louis may be said to be the founder of a school in which too exclusive importance is attached to experimental methods of investigation. He is called the author of a new system, the "*numerical system*," as it is termed. Had we time to enter into a discussion of the subject we should expect to show that his claims are not so much those of an *inventor*, as of a *reformer*. The *numerical system* can hardly be called a *discovery*, but it must be conceded to be an *improvement*, which, there is every reason to think, will prove invaluable in its useful services to science. The distinguishing features of the numerical system are briefly as follows:—It demands, in the first place, proper capacity and qualifications to *observe*, i. e. to ascertain palpable phenomena. To do this the individual must not only be well qualified in all the fundamental branches of medical science, but must have had some practice as an observer. Merely to observe well is not an intuitive gift, nor is it easily acquired. There are abundant liabilities to mistakes and oversights even in the exercise of the senses. Next to correct observation, the most important point is to collect *all* the phenomena relating to the case which is the subject of examination—not confining the attention simply to what are more prominent, but, also, being careful to note *every thing;* as it is equally important with reference to the results, to know with positiveness that certain phenomena are not, as that certain phenomena are present—in other words to acquire both positive and negative facts. The observations are to be carefully recorded. It is an essential feature of this system that the memory is not to be relied upon for the preservation of *data* for scientific purposes. It is owing to the non-observance of this precaution, in a great measure, that there is so much false experience in medicine. No man's powers of recollection are adequate to retain all the details involved in an extensive collection of medical observations. Having, in this way, collected a sufficient number of observations, they are finally to be analyzed, and compared. This means arranging them in juxtaposition in every possible mode, and determining the results by counting, i. e. in figures. These numerical comparisons will furnish facts available for every branch of medical science. For example, suppose we have complete histories of one hundred cases of typhoid fever. By pursuing the method just detailed, we find that certain symptoms are present in all cases; other symptoms are present in one-half, one-third, one-fourth, as the case may be. Now this enables us to give a perfectly accurate description of the disease, which is of incalculable benefit in diagnosis; especially when the results are compared with the history of other acute diseases, which have been investigated with the same rigorous method of analysis and comparison. A description based upon these data, contrasted with one presenting only the more prominent features, loosely observed, will be like the accuracy of a diagram determined by exact measurement, compared with scenic representations intended only for a distant effect. It will bear close inspection, may be relied upon with certainty, and will answer for all time. Next, as regards the pathological character of the disease. Certain lesions will be found, perhaps, to be constant, being present in every case; others are present in different but exact proportions to the whole number of cases. We have, then, valuable materials for pathological reasoning as to the true seat, nature, and tendencies of the disease. Next, as regards duration of the disease, and the influence of different methods of treatment, it is evident that much useful information is to be obtained from a series of observations carefully collected, and rigidly analyzed. We shall have occasion, from time to time, while treating of individual diseases, to adduce illustrations of this method of prosecuting experimental investigations. I will not, therefore, in this connection, devote farther time to its explanation. You will perceive, I think, on proper reflection, that it possesses great value, but that its extent of application is limited, and that it is absurd to say that it is to supersede rational deductions. It is, in fact, neither more nor less than a system of statistics. Now, is it to be supposed that we are to be directed, in all cases, in the conclusions and actions involved in the practical duties of our profession, by statistical laws? By no means. Admit, for argument's sake, that we possessed accurate statistical information on every practical point involved in any case of disease which may come under our care; grant, in the first place, that we have diagnosticated a disease; and that we are well acquainted with its history and pathological character—we wish to decide upon the treatment which it is best to pursue—we refer to our statistical tables, which we will imagine to be extensive and accurate. We learn therefrom that, with such and such treatment, the ratio of recoveries was greatest. Recollect, I am now supposing that every possible variety of treatment has been subjected, in a most perfect and rigorous manner, to numerical comparison—a supposition which is entirely gratuitous, for it is utterly impossible for statistics to arrive except at a remote approximation to such completeness. But grant, for the moment, that this has been attained, and that it has been demonstrated by precise, arithmetical computations, that a certain course of treatment will be successful in the greatest number of cases—Has it been successful in every case? No. And why? The only answer is, that these were exceptional cases. Now, how can it be proved that in some of these exceptional cases a different mode of treatment from that which has been mathematically shown to succeed best in the average, would not have been preferable? True, a certain course of therapeutics may save a greater *per centage* of lives than others, but does it follow from this that each individual patient out of the hundred, will do best under that course? By no means. If I have succeeded in making my argument clear, it seems to me it must be evident that statistical laws are not, as a matter of course, uniformly applicable to individual cases. A precise analogy obtains between the point under consideration, and the principle of life assurances. The insurer of lives bases his action upon statistics calculated from bills of mortality. He knows precisely what amount of money it is worth to guaranty one hundred persons, of a given age, duration of life, for a given period. This has been reduced to great precision in its application to a large number of individuals. But do we go to the life assurance offices to know how long, individually, we are to live? We could learn there how many out of one hundred will be likely to die within a certain period, but we should receive little insight into our own destiny in this particular. So it is with principles of therapeutics founded exclusively on statistics. We may determine what method of treatment will give a greater ratio of cures, but in treating individual cases we must consider, mainly, the particular circumstances of each case, and disregard these empirical laws, if they conflict with rational deductions. I do not mean, by this remark, that these laws are of but little practical use. They are in a high degree useful, but chiefly as auxiliary to reasoning from other data. I mean, in short, that empirical medicine, and rational medicine, are neither to be pursued to the exclusion of the other. They cannot be disjoined. Numerical experience is a means of developing truth, but it is still more important as a means of confirming, or disproving the results of ratiocination. Rational deductions, on the other hand, must often be our sole reliance, from the limited extent to which numerical laws are attainable; and the application of these laws must always involve reasoning and judgment.

In the latter portion of this discourse, Gentlemen, I have discursively touched upon topics which I would commend to your examination and reflection. The methods by which scientific truth is to be elicited, and discriminated: the sources of error—in fine, the principles of *medical logic*, are not only worthy of study, but are absolutely essential to success and eminence in the profession which you have chosen. In the foregoing few remarks I have only aimed to furnish some hints, with the hope that they may serve to give your attention and thoughts a proper direction. Did time permit, I should be glad to consider the subject at greater length—but, as it is, perhaps you have felt some impatience to enter upon the subjects strictly appertaining to medical instruction. Without farther preliminaries, except to consider somewhat the classification of diseases, and determine the arrangement which it it is best to adopt, we shall commence, at our next meeting the study of special diseases.

